# Polo-like kinase 4: the odd one out of the family

**DOI:** 10.1186/1747-1028-5-25

**Published:** 2010-09-29

**Authors:** James E Sillibourne, Michel Bornens

**Affiliations:** 1 Institut Curie, UMR144, 26 rue d'Ulm, 75248, Paris Cedex 05, France

## Abstract

Polo-like kinase 4 (PLK4) is a unique member of the Polo-like family of kinases that shares little homology with its siblings and has an essential role in centriole duplication. The turn-over of this kinase must be strictly controlled to prevent centriole amplification. This is achieved, in part, by an autoregulatory mechanism, whereby PLK4 autophosphorylates residues in a PEST sequence located carboxy-terminal to its catalytic domain. Phosphorylated PLK4 is subsequently recognized by the SCF complex, ubiquitinylated and targeted to the proteasome for degradation. Recent data have also shown that active PLK4 is restricted to the centrosome, a mechanism that could serve to prevent aberrant centriole assembly elsewhere in the cell. While significant advances have been made in understanding how PLK4 is regulated it is certain that additional regulatory mechanisms exist to safeguard the fidelity of centriole duplication. Here, we overview past and present data discussing the regulation and functions of PLK4.

## The structure of PLK4

PLK4 was initially identified in the mouse as a kinase sharing homology with *Drosophila *Polo kinase, *S. cerevisiae *CDC5 and murine Snk (it was subsequently named as *S*nk *a*kin *k*inase (SAK) due to its homology with the latter) [1]. Recent evolutionary studies have shed light onto the origins of PLK4/SAK, which appears to have arisen through gene duplication and subsequent subfunctionalization [2,3]. Homologues of PLK4 are present in most opisthokonts (organisms that have a single posterior flagellum), with at least one exception, the nematode *C. elegans*. This organism has no direct homolog of PLK4 although the kinase zyg-1 has been proposed to be a functional equivalent because it is essential for centriole duplication in the worm [4]. Interestingly, zyg-1 shares closer homology to the centrosomal kinases NIMA and MPS1 than to *C. elegans *Polo-like kinases (PLK1-3) strongly suggesting that it did not arise through duplication of the PLK gene [2].

**Figure 1:**
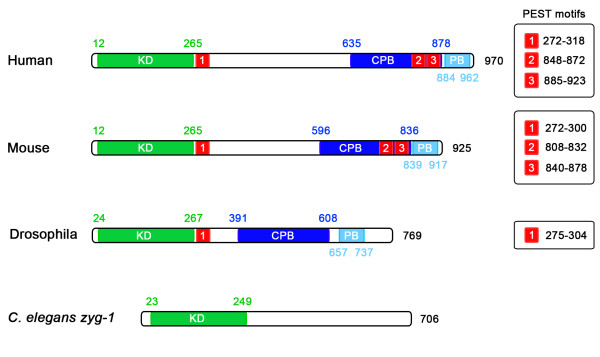
**The structure of PLK4 and zyg-1**. PLK4 differs from the other members of the PLK family in that it only has a single Polo-box, as opposed to two, and possesses a crypto Polo-box instead. These domains are involved in targeting the kinase to the centrosome and both are independently able to localize to the centrosome when fused to GFP. In *Drosophila *PLK4, a PEST sequence located after the catalytic domain is an important element controlling the stability of the kinase. This PEST sequence is also present and functional in mouse and human PLK4 along with two PEST sequences at the carboxy terminus of the kinase. The *C. elegans *kinase, *zyg1 *is also shown because it fulfills a similar role as PLK4 in the nematode although it is not related to it.

While the structure of PLK4, in terms of arrangement of its functional domains (Figure 1), is similar to that of the other members of the Polo-like kinase family there are several significant differences and consequently its sequence is more divergent in comparison [5]. Common to the PLKs is an amino-terminal catalytic domain, which contains the unique ATP-binding site Gly-X-Gly-X-Phe-*Ala*, as opposed to the Gly-X-Gly-X-X-*Gly *motif commonly found in kinases [6-8]. Sequence homology of the catalytic domain is highest in the first three members of the PLK family, with PLK1 sharing 53 and 54% identity with PLK2 and PLK3, respectively while it is lower for PLK4 which only shares 37% identity with PLK1 [9]. Predicting PLK4 phosphorylation sites has proven to be difficult because the kinase phosphorylates in a context-dependent manner, whereby residues surrounding the phosphorylation site influence the kinase's ability to phosphorylate it [10]. Three different groups have derived consensus phosphorylation motifs [9-11], all of which share some common elements (Table 1).

**Table 1:** PLK4 phosphorylation motifs

	-3	-2	-1	S/T	1	2	3	4
**Leung et al. (2007)**	-	Charged	I, L and V unfavoured		Hydrophobic (large)	Hydrophobic (large)	X	Charged or P

**Johnson et al. (2007)**	R/K	E/D	X		Hydrophobic/Y	Hydrophobic/Y	X	S/T/A

**Sillibourne et al. (2010)**	Aliphatic, hydrophobic or basic (small to medium)	X	Large residues unfavoured		Aliphatic (charged residues unfavoured)	Aromatic or aliphatic (large)	X	-

Three different groups have derived a PLK4 phosphorylation motif, while differences exist there are some common elements in all three. PLK4 has a preference for small to medium aliphatic or basic residues at the -3 position, aliphatic or charged residues at the +1 position and aromatic or large hydrophobic residues at the +2 position. The studies of Leung *et al *(2007) and Sillibourne *et al *(2010) show that large amino acids, in particular I, L and V, are unfavoured at the -1 position. There is a preference for a charged residue at the -2 position which is influenced by the residue in the +4 position and those outside of the motif. The interdependency between residues in the -2 and +4 positions, coupled with the influence of residues surrounding the phosphorylation motif, indicates that PLK4 is a context dependent kinase and renders the prediction of phosphorylation sites more difficult. Supporting this, it has been shown that a large number of predicted sites present in candidate PLK4 substrates, which fit the consensus phosphorylation motif well, are not phosphorylated by the kinase. All in all, this means that the identification of PLK4 substrates will remain a significant challenge for the future.

All members of the family of Polo-like kinases possess a characteristic Polo-box, a conserved 64 amino acid motif located at the carboxyl terminus of the protein, which not only dictates the substrate specificity of the kinase, but also regulates its function [12,13]. PLK1, 2 and 3 possess two Polo-box domains at their C-terminus, while PLK4 only has one [14]. In place of a second Polo-box, PLK4 possesses a larger crypto Polo-box domain that has weaker homology with the Polo-box domain [14,15]. The fact that PLK4 only possesses a single Polo-box has important implications for its regulation and substrate repertoire. PLKs 1 to 3 bind to proteins that have previously been phosphorylated via their tandem Polo-boxes, which form intramolecular heterodimers and recognize the sequence Ser-pSer/pThr-Pro-X [13]. Polo-box dimerization and binding to the phospho-motif is thought to regulate the activity of the kinase by inducing a change in its conformation, allowing the catalytic domain to have access to its substrate [13]. Because the Polo-box and crypto Polo-box of PLK4 do not form an intramolecular heterodimer, it has been suggested that PLK4 is not subject to the same form of regulation [14,15]. The PLK4 Polo-box does, however, homodimerize in an intermolecular manner and this may be involved in regulating PLK4 kinase activity [14]. The Polo-boxes of PLK1-3 are also important for targeting the kinases to particular subcellular sites and in this respect the Polo-box and crypto Polo-box of PLK4 serve a similar function. Both are independently able to localize to centrosome, when expressed in fusion with EGFP [14], and only when both are removed from PLK4 does it fail to localize to centrosome and its function is suppressed [14,16]. This suggests that the Polo- and crypto Polo-boxes of PLK4 are protein-protein interaction domains responsible for targeting the kinase to the centrosome although the identities of their binding partners remain to be discovered. The ability of the Polo-box and crypto Polo-box domains of PLK4 to bind to the centrosome could also be explained by the fact that both are able to self-associate with other domains within the kinase [14].

A further difference between PLK4 and the other PLKs is that it possesses a large central domain, which is conserved through evolution, although the function of this domain remains unknown [2].

PLK4 also possesses three PEST sequences, domains rich in proline (P), aspartate (D), glutamate (E), serine (S) and threonine (T) residues, which govern protein stability [1,17]. The first PEST sequence is conserved, being present in many species including, *H. sapiens*, *M. musculus*, *D. melanogaster*, *D. rerio *and *X. laevis *[18,19]. The function of these sequences in regulating the turn-over of PLK4 will be discussed in more detail later.

## Functions of PLK4

Studies carried out in knockout mice have demonstrated that PLK4 is essential for postgastrulative embryonic development and is required for mitotic progression [20]. PLK4-/- embryos arrest at stage E7.5 with increased numbers of apoptotic and late mitotic cells [20], while PLK4+/- embryos develop normally but have an increased incidence of spontaneous liver and lung cancers [21]. Partial hepatectomy experiments on PLK4+/- mice identified a defect in mitotic entry and exit, with cyclin B1 accumulation being delayed and prolonged for longer than normal [21]. Inspection of dividing hepatocytes from these mice showed that nearly one third had tripolar or tetrapolar spindles, which consequently led to the formation of disorganized liver tissue and an increased incidence of tumors [21]. Embryonic fibroblasts derived from PLK4+/- mice have supernumerary centrosomes, frequently undergo aberrant chromosome segregation and have a higher level of aneuploidy than wild-type mice [21].

At present, two mitotic PLK4 substrates have been identified: the phosphatase CDC25C [22] and the RhoA guanine exchange factor (GEF), Ect2 [23]. CDC25 was selected as a candidate PLK4 substrate based on the fact that this phosphatase is phosphorylated by PLK1 and PLK3 (raising the possibility that it is a common PLK substrate) and both CDC25C and PLK4 localize to the centrosome. Ect2 also seems to be a common PLK substrate, as it is phosphorylated by both PLK1 [24] and PLK4 [23]. Ect2 is a guanine exchange factor for the small GTPase RhoA and is required to activate it during cytokinesis to ensure correct positioning of the cleavage furrow [25]. Association of Ect2 to the central spindle is dependent upon PLK4 activity, as it fails to localize correctly in PLK4+/- MEFs. These cells frequently undergo cytokinetic failure because of the lack of Ect2 at the central spindle and insufficient RhoA activity [23].

## PLK4 and centriole duplication

The centrosome duplicates once per cell cycle [26] and PLK4 plays an essential role in this process [16,27]. Over-expression of PLK4 in somatic cells results in the excessive formation of centrioles [16], the core structures of the centrosome, and in *Drosophila *oocytes the de novo formation of centrioles [28]. Conversely, depletion of PLK4 by RNAi prevents centriole duplication [27], causing mitotic defects and in some cell lines it can induce apoptosis [29].

**Figure 2:**
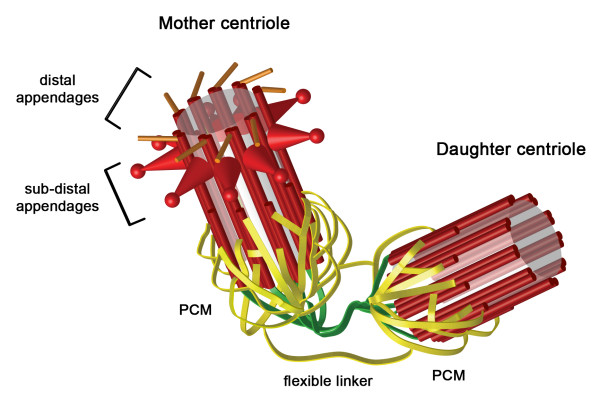
**The centrosome**. The centrosome consists of two centrioles that are formed from nine sets of microtubules (red tubes), which are triplet at the proximal ends and doublet at the distal ends of centrioles. The two centrioles attached to one another via their proximal ends by a flexible linker (green ribbons). Surrounding the proximal ends of each centriole is a matrix of proteins, the pericentriolar material (PCM) that is a site of microtubule nucleation as well as procentriole assembly (yellow ribbons). The two centrioles differ from one another, as one is slightly longer and also possesses two sets of appendages (distal and sub-distal drawn as orange sticks and red cones, respectively). This centriole is referred to as the mother while the other is the daughter centriole. PLK4 localizes to the proximal ends of both centrioles and the distal end of the mother centriole.

A centrosome consists of two centrioles [26], barrel-shaped microtubule-based structures, which are connected at their proximal ends by a flexible linker [30,31] (Figure 2). The two centrioles differ from one another, as one is slightly longer and possesses two sets of appendages at its distal end (sub-distal and distal appendages) [31,32]. This centriole is referred to as the mother and the other as the daughter centriole. The proximal ends of each centriole are surrounded by a matrix of proteins, referred to as pericentriolar material (PCM), which serves as a site of microtubule nucleation [26,32]. The PCM also serves to create environment favourable for the assembly of procentrioles [33], nascent centrioles, which form orthogonally from the existing centrioles [26,32,34]. Centriole duplication, similar to DNA replication is licensed to occur once per cell cycle [35,36] and procentriole assembly starts at the G1/S border [34,37]. Procentriole assembly begins with the formation of a cartwheel structure to which microtubules are attached and elongated during the course of the cell cycle. The initial steps of procentriole assembly are dependent upon several proteins including SAS-6 [38], Cep135 [39,40], SAS-4 (CPAP) [41,42], γ-tubulin [43] and CP110 [44] as well as PLK4 [16,27]. At present, the centriolar substrates of PLK4 remain to be identified although SAS-6 is a possible candidate as work carried out in *C. elegans *has shown that zyg-1-dependent phosphorylation of SAS-6 is required for procentriole formation [45]. Other factors are also required and include an array of kinases, such as PLK1 [46], PLK2 [47], cyclin-dependent kinase 2 (Cdk2)/cyclin A/E [48,49] and Mps1 [50] as well as the phosphatase Cdc14B [51]. Later on in the cell cycle, during mitosis, procentriole elongation is completed [37] and proteins found in mature centrioles, such as the distal lumen protein hPOC5 [52], become incorporated into the procentriole structure. Throughout the duplication process, each procentriole remains closely associated with its parental centriole and this tight association (referred to as engagement) is a key aspect of the licensing mechanism [34,35]. It prevents centriole reduplication and it is only during late mitosis that it is lost [53]. Centriole disengagement allows a new round of centriole duplication to occur in the next cell cycle and thereby acting as a licensing mechanism. Disengagement is dependent upon the activity of separase, a protease involved in breaking sister chromatid cohesion in mitosis and PLK1 [46].

Immunoelectron microscopy has shown that myc-tagged PLK4 localizes to the outer wall of centrioles and seems to be enriched at the proximal ends [43]. This localization is consistent with PLK4's role in centriole duplication because it is next the site of procentriole formation. However, PLK4 has also been observed, by immunofluorescent microscopy, at the distal end of the mother centriole close to the sub-distal and distal appendages [11]. The exact function of PLK4 at the distal end of the mother centriole remains to be elucidated, but it may be involved in centriole maturation including, centriole elongation and/or appendage assembly.

## PLK4 abundance and activity during the cell cycle

PLK4 abundance must be tightly controlled to ensure that centriole duplication goes according to plan, as either too little or too much of the kinase can have a deleterious effect upon the fidelity of centriole duplication. Too much PLK4, as demonstrated by over-expression of the kinase, overrides the centriole licensing mechanism and results in centriole amplification with multiple procentrioles forming around each parental centriole [16,27]. An insufficient amount of PLK4 may also give rise to the formation of abnormal centrioles and microtubule-based structures. In HCT116 cells microtubule-based γ-tubulin-containing structures lacking key centriolar components such as SAS-4/CPAP, SAS-6, and Cep135 have been observed [54]. These structures are commonly formed of microtubule bundles and some resemble centrioles, but lack a large number of centrosomal proteins and are unable to nucleate microtubules. Importantly, the incidence of these structures is reduced upon expression of PLK4 suggesting that there formation is due to insufficient kinase activity. Supporting these data, embryonic fibroblasts derived from PLK4 heterozygous mice exhibit an increased incidence of supernumerary centrosomes, which may, in fact, reflect the formation of abnormal microtubule-based structures [21].

**Figure 3:**
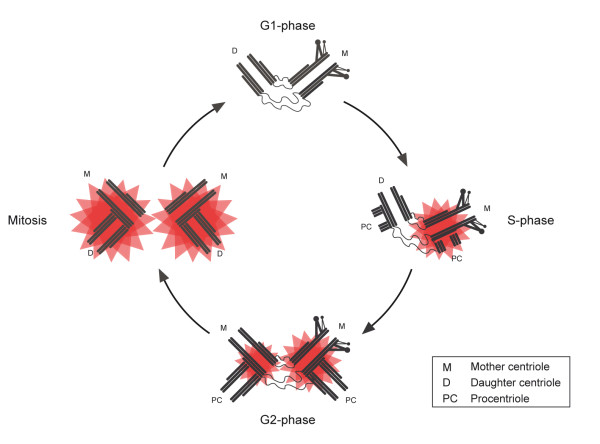
**Centriole duplication and temporal activation of PLK4**. In G1, PLK4 is present at centrioles but active kinase cannot be detected indicating that PLK4 is inactive at this point in the cell cycle. At the G1/S boundary centriole duplication begins with the formation of procentrioles at the proximal end of each parental centriole. This coincides with activation of PLK4 at the mother centriole (active kinase drawn as a red star). PLK4 becomes active at the replicating daughter centriole later on in the cell cycle in G2. By mitosis both centrosomes possess a similar amount of active PLK4 and procentriole elongation has been completed.

Several studies, carried out in human and Drosophila cell lines, have shown that PLK4 abundance at the centrosome fluctuates during the cell cycle [11,18,19]. In the case of Drosophila S2 cells, PLK4 is almost undetectable in interphase, but is clearly detectable during mitosis where its levels are at their highest [18,19]. In cultured human cells, PLK4 levels at centrosomes follow a similar trend, with levels being low in G1 and increasing incrementally from S phase onwards to reach a maximum in mitosis [11]. While these data suggest that high levels of PLK4 are required during mitosis they do not give any indication of the kinase's activity. One study took advantage of the fact that PLK4, like many kinases, autophosphorylates upon activation [11]. Several potential autophosphorylation sites were identified and one of these, S305, was found to be phosphorylated in cultured cells. By raising a phospho-specific antibody against one of these sites, S305, it was possible to determine when PLK4 became active in the cell cycle. This revealed a number of remarkable findings. Firstly, PLK4 is present at centrioles in G1, but S305 phosphorylated PLK4 is undetectable suggesting that the kinase is inactive at this point in the cell cycle (Figure 3). PLK4 first becomes active in S phase and the amount of active kinase approximately doubles at each cell cycle transition to reach a maximum in mitosis. Secondly, there is a delay in the activation of PLK4 at the replicating daughter centriole, with PLK4 first becoming active at the replicating mother centriole in S phase and then later at the replicating daughter centriole in G2. Thirdly, more active PLK4 is associated with the replicating mother centriole than the replicating daughter throughout interphase although by mitosis parity is reached. These results support the proposal that centriole may be initiated at the mother centriole first and then later at the daughter centriole. Lastly, it has been shown that active PLK4 is restricted to the centrosome, which may serve as a mechanism preventing centriole formation elsewhere in the cell and ensure that de novo centriole formation does not occur.

## The regulation of PLK4 stability

**Figure 4:**
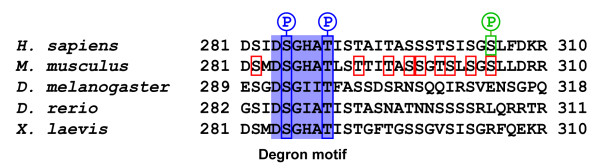
**Regulatory phosphorylation sites in PLK4**. The degron motif of PLK4 (highlighted in blue) is conserved with phosphorylation of its serine and threonine residues creating a binding site for the F-box protein β-TrCP, which forms part of the SCFβ-TrCP ubiquitin ligase complex. Upon SCFβ-TrCP binding, PLK4 is subsequently ubiquitinated and targeted to the proteasome for degradation. The identity of the kinase responsible for phosphorylating the two residues in the degron motif is currently unknown. Autophosphorylation plays a role in controlling the stability of PLK4 and it has been shown that the region spanning residues 282 to 305 of *M. musculus *PLK4 is heavily autophosphorylated. The precise identities of the residues autophosphorylated are not known and only potential sites can be proposed (marked in red). One of these sites, S305, is conserved and is autophosphorylated in *H. sapiens *PLK4 (marked in green), although it has no direct role in regulating the turn-over of the kinase directly because its mutation to an alanine does not increase the stability of the kinase. However, it does seem to play a role in centriole duplication with its mutation to a glutamate increasing the incidence of centriole amplification in PLK4-overexpressing cells.

PLK4 is a short-lived protein, with a half-life of between 2 to 3 hours, which is ubiquitinylated and degraded by the proteasome [1,55]. The stability of the kinase is governed by three PEST sequences [1], one within the amino-terminus and two within the carboxy terminus of the kinase, and internal deletion studies have shown that all of them play a part in controlling the turn-over of the kinase [8]. Deletion of the first PEST sequence, however, stabilizes the kinase more than deletion of the two carboxy-terminal PEST sequences, suggesting that it has a greater influence in controlling PLK4 stability [8]. The first PEST sequence of PLK4 contains a degron motif, DGSXXT, which is conserved through evolution being present in *H. sapiens*, *M. musculus*, *D. melanogaster*, *D. rerio *and *X. laevis *PLK4 (Figure 4) [18,19]. Phosphorylation of the serine and threonine residues in the degron motif generates a binding site for the ubiquitin ligase complex, Skp1/cullin/F-box (SCF). The SCF consists of cullin 1, a scaffolding protein that binds two invariable proteins Skp1, Rbx and a variable F-box protein, which determines the target specificity of the complex [56,57]. The SCF ubiquitin ligase complex has previously been implicated in centriole duplication. The *Drosophila slimb^crd ^*(centrosome replication defective) mutant, which has a P-element insert within the 5' untranslated region of *slimb*, a F-box protein, possesses supernumerary centrosomes although not all of the centrosomes that form are mature and able to nucleate microtubules [58]. There is also evidence indicating that mammalian *slimb *(β-TrCP) is involved in regulating centriole duplication. Embryonic fibroblasts derived from β-TrCP -/- mice have an increased incidence of supernumerary centrosomes compared to wild-type MEFs although this may be due, in part, to a mitotic defect [59].

Upon binding to the phosphorylated degron motif the SCF ubiquitinates PLK4 and it is targeted to the proteasome for destruction [18,19]. Two different approaches were employed to demonstrate a specific role for the SCF^slimb/β-TrCP ^complex in regulating the turnover of PLK4. The first involved disrupting the SCF^slimb/β-TrCP ^complex by siRNA-mediated depletion of either the cullin or *slimb*/β-TrCP subunits. The second approach involved mutation of the serine and threonine residues within the degron motif of PLK4 to alanine, to prevent phosphorylation and generate a stabilized form of the kinase that was no longer recognized by the SCF^slimb/β-TrCP ^complex. Both of these approaches resulted in elevated levels of centriole amplification compared to control siRNA depletions or over-expression of the wild-type form PLK4. These results led to the proposal that the increased incidence of centriole amplification is directly attributable to the higher expression level of PLK4 [18,19]. Mutation of the degron motif to prevent phosphorylation clearly stabilizes PLK4, but fluorescence intensity measurements have shown that there is no difference in the amount of over-expressed PLK4 at the centrosomes of wild-type or degron-mutated PLK4-transfected cells exhibiting centriole amplification. Furthermore, mutation of the degron motif appears to promote an increase in the amount of active PLK4 because a greater proportion of the kinase is S305 autophosphorylated [11]. These results suggest that the higher incidence of centriole amplification observed in cells expressing degron-mutated PLK4, compared to wild-type, is due to an increase in the amount of active kinase.

At present the kinase responsible for phosphorylating the degron motif is unknown but there is mounting evidence indicating that autophosphorylation plays a role in regulating the stability of the kinase. A link between PLK4 autophosphorylation and kinase stability was first established when it was observed that mutation of the kinase domain, to render the kinase catalytically inactive, resulted in increased expression of the kinase compared to wild-type [8]. A 23 amino acid region beyond the catalytic domain in mouse PLK4, which encompasses the degron motif, is heavily autophosphorylated and the deletion of this entire region vastly increases its stability and ability to trigger centriole amplification [60]. Similarly, mutation of the serine and threonine residues to alanine in this region stabilizes the kinase and augments its ability to amplify centriole number [60]. A more recent paper has elegantly shown that PLK4 autophosphorylation occurs in *trans*, where the molecules in the dimer phosphorylate each other, and confirmed that autophosphorylation is necessary to target the kinase for degradation [61]. This finding also explains why inducible cell lines stably transfected with catalytically inactive PLK4 are able to trigger centriole amplification upon induction of gene expression. Catalytically inactive PLK4 dimerizes with the endogenous kinase, but is unable to phosphorylate it, which effectively protects the endogenous kinase from degradation because the SCF^β-TrCP ^complex cannot bind to it. As the endogenous kinase in no longer under the control of this ubiquitin-mediated degradation pathway centriole amplification ensues [61].

Where SCF-mediated ubiquitination takes place in the cell has yet to be determined conclusively. Components of the SCF including Skp1 and culllin 1 have been found localize to the centrosome throughout the cell cycle [62,63]. Cullin 1 seems to be enriched on the mother centriole [64], where there is more PLK4 and centriole duplication is probably first initiated [65], suggesting that there may be a greater demand for SCF activity at this site to prevent centriole amplification.

While it is clear that the SCF^β-TrCP ^ubiquitin ligase complex has an important role in targeting PLK4 to the proteasome for degradation, it is not the only factor controlling the kinase's turn-over and stability. *Drosophila *PLK4 SCF^slimb^-binding mutants that can no longer be phosphorylated on the degron motif are still subject to degradation in G2 phase of the cell cycle [19]. The introduction of similar mutations in mouse PLK4 still results in degradation of the kinase. It is possible that in the absence of SCF^β-TrCP ^activity the APC/C ubiquitin ligase complex may take over, as this ubiquitin ligase has been proposed to be involved in regulating PLK4 degradation before [66].

## Alternative forms of regulation

The stability of PLK4 may be governed by alternative mechanisms such as the phosphorylation-dependent stabilization of PLK4 by other kinases and there are data to suggest this is the case. Yamashita et al demonstrated that phosphorylation of a tyrosine residue in the N-terminus of PLK4 by the kinase Tec increased the stability of PLK4 and promoted PLK4 autophosphorylation [8]. The Tec-dependent increase in PLK4 stability is interesting because it suggests that phosphorylation by other kinases may play a role in governing its turn-over. Such a mechanism might be at work at centrosome and it could result in the local stabilization of PLK4 at this site. In support of this it has been shown that S305 autophosphorylated PLK4 at centrosomes exhibits a similar shift in mobility as Tec phosphorylated PLK4 [11].

Another possible regulatory mechanism could be proposed from work carried out on the *C. elegans *centrosomal kinase, zyg-1. In a screen for suppressors of the zyg-1(it25) temperature-sensitive mutant allele a number of candidate genes were identified including *s*uppressor of *zy*g-1 20 (*szy-20*) [67,68]. This gene encodes an RNA-binding protein that localizes to the centrosome and appears to negatively regulate the abundance of zyg-1 [68]. Szy-20 is a conserved and it will interesting to see if the vertebrate homologues of this protein are involved in controlling PLK4 abundance at centrosomes.

## Transcriptional control of PLK4

While much attention has been focused on proteasome-mediated degradation of PLK4 it is important not to overlook the fact that the PLK4 gene is transcribed in a cell cycle-dependent manner. PLK4 transcript levels are undetectable in G0, low in G1 and progressively increase through S and G2 to reach a maximum in mitosis [66]. It seems that PLK4 protein levels mirror those of PLK4 mRNA suggesting that gene transcription has a significant impact on controlling the overall expression level of PLK4. At present, little is known about the transcription factors controlling expression of the PLK4 gene. One report has shown that expression of the human PLK4 gene can be suppressed by the tumour suppressor p53 and this is dependent upon the activity of histone deacetylases (HDACs) [29].

## Conclusions

PLK1, PLK2 and PLK4 act in concert to control the licensing and duplication of centrioles and centrosome maturation. PLK4 represents a separate branch of the PLK family because it shares little homology with its other members as a result of rapid divergence through evolution. Its function in controlling centriole/basal body duplication is a result of sub-functionalization after duplication of the PLK gene. Before the innovation of PLK4, basal body duplication was probably under the control of a single PLK, although there are some ciliated species that do not have a PLK gene.

PLK4's role in centriole duplication is essential yet many questions remain to be answered. At present, no centriolar PLK4 substrates have been identified although one possible substrate is SAS-6, as work in *C. elegans *has shown that this protein is phosphorylated by zyg-1. The identification of PLK4 substrates should help to delineate the centriole duplication pathway and determine whether the kinase acts just once, to initiate duplication, or at multiple stages during the duplication process. It will also be important to identify the protein responsible for anchoring PLK4 to the centrosome via its crypto Polo and Polo-box domains. As PLK4 localizes to the proximal ends of and along the walls of centrioles it seems likely that PLK4 will interact with multiple proteins at the centrosome.

Determining how SCF^β-TrCP ^ubiquitin ligase-mediated degradation of PLK4 is coordinated and influenced by other factors during the cell cycle is crucial to understand how PLK4 levels are maintained within a certain threshold during the cell cycle. Clearly, if the threshold is crossed and PLK4 levels rise above normal the consequences can be catastrophic, particularly during centriole duplication because it overrides the licensing mechanism and multiple procentrioles form at each parental centriole.

The recent discovery that PLK4 is involved in cytokinetic exit broadens the role of this kinase beyond centriole duplication and demonstrates that the kinase has multiple functions in the cell. Several lines of evidence support a role for PLK4 in mitotic progression, including the identification of mitotic substrates such as CDC25C and Ect2, the delayed entry of PLK4+/- dividing hepatocytes into mitosis coupled with persistently elevated levels of cyclin B1 and the fact that active PLK4 levels reach a maximum during mitosis. This suggests PLK4 is linked to cell cycle regulators, but also raises the possibility that the kinase is involved in regulating centrosome maturation, such as procentriole elongation, which is completed in early mitosis, or the transformation of the daughter centriole into a mother centriole (appendage formation). It is clear that much work remains to be done before we fully understand the functions of this kinase in the cell.

## Competing interests

The authors declare that they have no competing interests.

## Authors' contributions

JES made the figures and drafted the manuscript. MB read and corrected the manuscript. All authors read and approved the final manuscript.
